# Systematic Review of the Role of Stereotactic Radiotherapy for Bone Metastases

**DOI:** 10.1093/jnci/djz101

**Published:** 2019-05-23

**Authors:** Katie L Spencer, Joanne M van der Velden, Erin Wong, Enrica Seravalli, Arjun Sahgal, Edward Chow, Jorrit-Jan Verlaan, Helena M Verkooijen, Yvette M van der Linden

## Abstract

**Background:**

Stereotactic radiotherapy (SBRT) might improve pain and local control in patients with bone metastases compared to conventional radiotherapy, although an overall estimate of these outcomes is currently unknown.

**Methods:**

A systematic review was carried out following Preferred Reporting Items for Systematic Reviews and Meta-Analyses guidelines. Pubmed, Embase, and Cochrane databases were systematically searched to identify studies reporting pain response and local control among patients with bone metastases from solid-organ tumors who underwent SBRT in 1–6 fractions. All studies prior to April 15, 2017, were included. Study quality was assessed by predefined criteria, and pain response and local control rates were extracted.

**Results:**

A total of 2619 studies were screened; 57 were included (reporting outcomes for 3995 patients) of which 38 reported pain response and 45 local control rates. Local control rates were high with pain response rates above those previously reported for conventional radiotherapy. Marked heterogeneity in study populations and delivered treatments were identified such that quantitative synthesis was not appropriate. Reported toxicity was limited. Of the pain response studies, 73.7% used a retrospective cohort design and only 10.5% used the international consensus endpoint definitions of pain response. The median survival within the included studies ranged from 8 to 30.4 months, suggesting a high risk of selection bias in the included observational studies.

**Conclusions:**

This review demonstrates the potential benefit of SBRT over conventional palliative radiotherapy in improving pain due to bone metastases. Given the methodological limitations of the published literature, however, large randomized trials are now urgently required to better quantify this benefit.

Patients with advanced cancer commonly present with pain, with bone metastases being the most frequent cause of cancer-related pain (1,2). Conventional radiotherapy (cEBRT) is the cornerstone of bone metastases management. Approximately 60% of patients will experience pain relief following cEBRT, with 25% having complete resolution at the treated site (3,4). The mean duration of palliation is approximately 4 months (5,6). To date, no dose-response effect has been demonstrated; a single 8 Gy dose provides equivalent pain control to more fractionated regimens of 20–30 Gy (3,4). cEBRT is routinely delivered using single- or parallel-opposed fields. More recently, advances in the conformality of image-guided radiotherapy techniques, such as stereotactic body radiotherapy (SBRT), have enabled the delivery of potentially ablative radiation doses while respecting healthy tissue constraints. Although established for treatment of small volume brain metastases (7), its role in bone metastases remains under investigation. It is hypothesized that the delivery of ablative doses to bone metastases may improve rates of pain response and duration, alongside greater local control (8). Several reviews have been published reporting the feasibility and efficacy of SBRT for bone metastases (8–11). No formal systematic review exists to assess relevant clinical outcomes (12,13). This study provides a systematic review, assessing pain response and local control following SBRT for bone metastases.

## Methods

This systematic review was carried out in line with Preferred Reporting Items for Systematic Reviews and Meta-Analyses guidelines and the Meta-analysis of Observational studies in Epidemiology checklist (12,13). The protocol was published in the PROSPERO international prospective register of systematic reviews (14).

### Search Strategy

A structured search was conducted in PubMed, Embase, and Cochrane electronic databases on March 16, 2016 (no initial time limit was applied). The search was updated on April 14, 2017. Reference lists from included articles were cross-checked to identify additional articles. For exact search terms used, please see the Supplementary Methods (available online).

### Study Inclusion Criteria

All original studies published in English, with full text available, reporting pain response (PR) or local control (LC) following SBRT to bone metastases from solid-organ malignancies, using 1 to 6 fractions, were included. Non-randomized studies were included. Studies could include patients with or without prior history of radiotherapy or surgery. Low-dose SBRT treatments could not be excluded because outcomes were not reported separately. All studies were independently assessed by two authors (JVDV, EW, or KLS) for eligibility based on their title and abstract. Where uncertainty remained, full-text was reviewed. Where individual patients were included in multiple published series, the most complete or recent article was cited (15). If less than 10 patients overlapped, both study populations were included.

### Data Extraction

The outcomes of interest were PR and LC reported at patient level. The definition of PR was that used in the original study. For every study, it was recorded whether the response was reported on a patient or lesion level. If available, the proportion of responders was recorded or calculated for assessable patients (patients with follow-up data available) and for the total treated population (TTP) (all patients originally included in the study regardless of availability of follow-up data). If the study was ambiguous regarding the results, a conservative assumption of assessable patients was made. Response definitions, response time point, adjustment for analgesia, how PR was collected (eg, by clinician or patient, 0–10 point numerical rating scale [NRS], visual analogue scale [VAS]), baseline patient and primary tumor characteristics, treatment dose, and fractionation were extracted for all included studies. As the majority of studies reported Karnofsky performance score (KPS), if performance status (PS) was reported as World Health Organization or Eastern Cooperative Oncology Group PS, it was converted to the KPS (16). Secondary endpoints were duration of pain relief, toxicity, and quality of life (17). Vertebral compression fracture rates were not assessed because they have been considered elsewhere (18). All data were extracted by both JVDV and KLS independently from the text or calculated using available information. Study authors were contacted for additional data if information was missing. The overall survival (OS) of the study population was extracted.

### Study Quality Assessment

Study quality was assessed using predefined criteria reflecting the Strengthening the Reporting of Observational Studies in Epidemiology checklist for reporting observational studies and incorporating key elements relating to the study question (19). The risk of bias was presented graphically as a proportion of all included studies. Study elements considered included whether the manuscript reported a clear definition of the study population (including baseline characteristics); if a clear definition of the SBRT technique was provided (including immobilization, imaging, volume definition, and dosimetric parameters); if the unit of response assessment was reported (ie, at patient or lesion level); if the time point for response assessment was provided; and whether response was reported for assessable patients or the TTP (including all treated patients).

In addition, criteria relevant to the assessment of PR for bone metastases were assessed. Specifically, the response definition was assessed relative to the international consensus on palliative radiotherapy endpoints (ICPRE), including whether adjustment for analgesia was made, if pain was reported by patients using a 0–10 point NRS, and how response was defined using this information (20).

### Quantitative Synthesis

The original protocol submitted to PROSPERO included a planned meta-analysis (14). The systematic review revealed marked clinical and methodological diversity with risk of bias in the included studies, making quantitative synthesis inappropriate. Meta-analysis outcomes are therefore not reported.

## Results

### Search Results and Overall Outcomes

**Figure 1.**
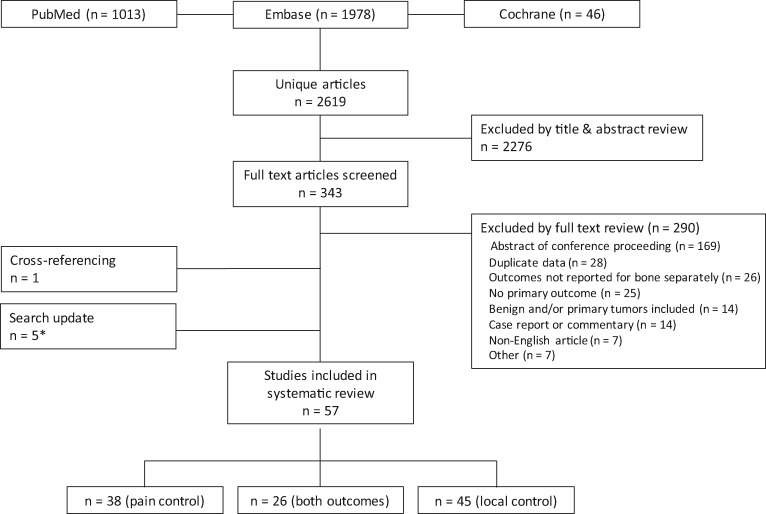
Flow diagram illustrating the searches, screening, and included number of studies. *Two of these five studies reported updated outcomes from previously included studies.

The initial search yielded 2619 articles. After screening of these articles on title and abstract, 343 studies proceeded to full-text screening, of which 290 were excluded (Figure 1). Exclusions were predominantly of conference proceedings, duplicate data, or SBRT case-series where outcomes following bone metastasis treatment were not reported separately. One additional article was included after cross-referencing because it used “high dose” instead of “stereotactic radiotherapy” in the title (21). The search update in 2017 yielded five more articles (21–26), of which two articles provided updated information, replacing the earlier included studies (24,27).

A total of 57 studies (reporting outcomes for 3995 patients) were included in the overall review. PR outcomes were reported in 38 (at least 2185 patients and 2947 lesions) and LC in 45 (at least 3455 patients and 4683 lesions). Both outcomes were reported in 26 studies. Patient and lesion numbers are not certain because of reporting limitations in the included studies. Only five included PR studies (28–32) and seven LC studies (21,22,29–33) reported outcomes for more than 100 patients. The median numbers of patients per study were 47 (PR) and 44 (LC). The included studies are detailed in Supplementary Tables 1 and 2 (available online).

In the TTP analysis, the range of PR outcomes varied from 27% (n = 11) (34) to 98% (n = 52) (35). LC rates at 1 year ranged from 25% (n = 28 patients) to 95.5% (n = 64 lesions) (36,37). The extremes of these ranges represent smaller studies. See Supplementary Tables 1 and 2 (available online) for details.

### Risk of Bias

One PR study reported outcomes from a randomized phase II trial (38), and two were non-randomized phase I–II trials (28,39). Seven PR studies reported a prospective design (31,37,40–44). The remaining 28 PR studies (73.7%) were retrospective cohort studies, or the study design was not clearly reported. Nine LC studies were carried out prospectively (31,33,37,40,45–49).

**Figure 2.**
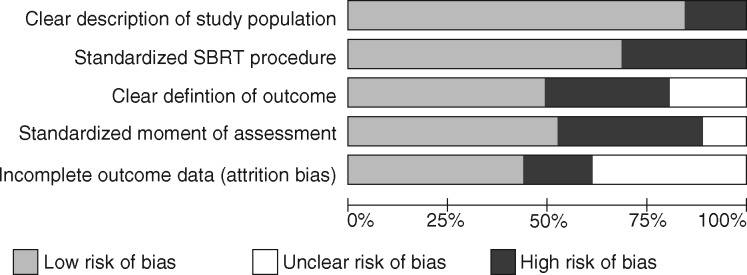
Risk of bias in the included studies. For each element the proportion of studies with high, low, and unclear risk of bias is illustrated. SBRT = stereotactic body radiotherapy.

Figure 2 shows the risk of bias in various elements of the study design and reporting. High risk of bias or unclear risk was particularly marked in outcome definition, outcome timing, and incomplete data availability. Detailed consideration of the study elements, which may be contributing to risk of bias, and their association with PR and LC, are included below.

### Study Populations

A majority of studies included patients with various primary cancer diagnoses (29 of 38 PR studies, 35 of 45 LC studies). The remaining studies focused on an individual diagnostic group: hepatocellular carcinoma (HCC; TTP PR = 64%–92% and TTP LC = 25%–79%) (36,50), melanoma (TTP LC = 42%) (51), breast (PR in assessable patients = 100%) (52), prostate (TTP PR = 83%–92%, LC = 96%–70%) (37,53), and renal cell cancer (RCC; TTP PR = 78%, LC = 74%–94% at 1 year) (22, 54–58).

In a majority of studies, radiotherapy was delivered to bone metastases located in the spine (32 of 38 PR and 38 of 45 LC). Only eight studies included other sites of disease, with TTP PR rates of 60%–88% and LC rates of 70%–96% (37,38,40, 53,55,56,59,60).

A large proportion of studies included patients with known malignant spinal cord compression (MSCC) (17 of 38 PR studies and 21 of 45 LC studies). The extent, however, was not always clear in the study report (where reported, this ranged from 20% to 47% of treated lesions). Outcomes were not reported separately, with only regression model results presented. For example, Lee et al. reported that patients with MSCC at baseline were more likely, on multivariable analysis, to experience pain recurrence after initial relief (hazard ratio [HR] = 10.15, 95% confidence interval [CI] = 2.82 to 36.22, *P* = .001) (61). Al-Omair et al. reported LC at 1 year of 84% for a cohort of individuals, predominantly with MSCC, undergoing surgical resection and subsequent SBRT (45). Studies excluding patients with MSCC reported LC of 82%–97% (24,54,57,60, 62), and TTP PR rates of 60%–92% (30,31,38–41,49,53,54,56,57,62–64). Anand et al. reported 95% PR in patients with epidural compression and 100% in those without. In this study, PR was defined as improvement on a 0–10 point VAS of more than 50%, no adjustment was made for analgesia, and no time point was specified. No statistically significant difference in LC at 1 year was seen (94% in patients with epidural compression and 83% without [*P* = .35, n = 76 lesions]) (35).

Wide variation was seen in the OS of the study populations (median survival in PR studies ranging from 8 to 30.4 months, and 8 to 47 months in LC studies; data not shown) (29,36,38,39,60). This is likely to reflect the variation in baseline characteristics of the study populations. For example, some studies excluded patients with predicted short survival or poor PS (37,50) either at the point of clinical decision making or retrospectively based on lack of follow-up data (65). Lee et al. demonstrated that poor PS is a predictor of pain recurrence post response on multivariable analysis (PS ≥ 2 vs <2, HR = 5.58, 95% CI = 1.37 to 22.60, *P* = .01) (61). Likewise, Park et al. reported better PS and lack of visceral metastatic disease (*P* = .004 and *P* = .001, respectively) to be predictive of treatment site-specific survival on univariate analysis (66).

### Treatment

Stereotactic dose schedules were standardized in 40 out of 57 studies reporting a reproducible treatment protocol, with dose schedules ranging from 6 Gy to 52.5 Gy in 1–6 fractions (Supplementary Tables 1 and 2, available online). Treatment volumes were defined in 39 studies in which the treatment planning margins varied from 0 to 5 mm. A simultaneous integrated boost approach was used in four studies (35,39,46,63).

Heron et al. (30) reported no difference in PR between fractionated (mean biologically effective dose [BED] 35.7 Gy) and single fraction (mean BED 43.2 Gy) SBRT (70% vs 71% at 1 year) in a mixed diagnosis population, although pain progression was more frequent following fractionated treatments. LC at 2 years was lower following single fraction treatment (96% vs 70%, respectively, *P* = .001) with higher retreatment rates (n = 153 lesions). The cohorts differed statistically significantly, however, and no adjustment was carried out for neurological symptoms (higher in the fractionated cohort), lesion volume (larger in the fractionated cohort [mean 81 vs 35 cm^3^]) and primary diagnosis (higher proportion of RCC and melanoma in the single fraction cohort). Lee et al. (50) also reported no statistically significant difference in PR or LC between treatments delivered using 1–4 fractions vs 10 fractions in patients with HCC, although for both outcomes the hypofractionated group experienced superior outcomes (n = 36 lesions). Similarly, Ryu et al. (42) reported no statistically significant difference in PR with SBRT dose (<14 Gy vs ≥14 Gy) in a mixed diagnosis population, although a strong trend toward increased PR was reported with dose no less than 14 Gy. The lack of numerical results means it is not possible to comment on the magnitude of this possible effect.

Specifically with regard to LC, Yamada et al. (24), in the largest series included here (n = 811 lesions), reported a statistically significant relationship between low (median = 17.1 Gy) and high (median = 23.6 Gy) D95 to gross tumor volume (GTV) and planning target volume with cumulative incidence of failure at 48 months (for GTV, failure rates of 14% [95% CI = 4.7% to 23.0%] vs 2.1% [95% CI = 1.0% to 3.2%], *P* < .001 on univariate analysis). No adjustment for treatment volumes or dosimetry was made. Bishop et al. (33) demonstrated greater LC with higher dose to tumor on multivariable analysis (GTV BED D_min_≥33.4 Gy [14 Gy in 1 fraction equivalent], HR = 0.29, 95% CI = 0.14 to 0.60, *P* = .001, n = 332 lesions). The number of patients receiving this lower dose was small (n < 21). Laufer et al. (67) demonstrated improved LC with high-dose SBRT (median dose 27 Gy in 3 fractions [competing risks sub-distribution hazard ratio (SHR) = 0.12, *P* = .04] or 24 Gy single fraction [SHR = 0.45, *P* = .09]) compared to low dose SBRT (30 Gy in 5–6 fractions, SHR = 1) but no relationship between fractionation and outcomes for high-dose treatments (n = 186 lesions). Ursino et al. (60) reported no association between dose and LC (n = 40 lesions) (60). Similarly, Choi et al. (68) reported single-session equivalent doses of less than 15 Gy_10_ were associated with increased local failure rates in patients undergoing re-irradiation within 1 year of prior treatment (*P* = .002, n = 21 lesions).

Two PR (43, 49) and two LC studies (45, 67) reported outcomes for a cohort of patients undergoing combined modality treatment with surgery and subsequent SBRT. TTP PR was reported to be 86% by Gerszten et al. (31), whereas LC was reported by Al-Omair et al. (45) and Laufer et al. (67) to be 84% in assessable patients. A further 16 of 38 PR studies and 19 of 45 LC studies included variable proportions of patients who underwent surgery prior to SBRT. Bate et al. (69) reported LC at 1 year of 95.8% following SBRT alone and 90.5% following combined SBRT and surgery. Staehler et al. (57) reported LC of 94% (95% CI = 85% to 90.4%) at 1 year in a patient cohort with RCC on concurrent systemic therapy. Reporting of both prior surgical decompression and concurrent systemic therapy was variable, and outcomes were rarely reported for these groups separately. As such, it is not possible to draw conclusions about the impact of combined modality therapy or concurrent systemic therapy.

A limited number of studies reported outcomes for only those patients known to have received prior radiotherapy. Thibault et al. (70) reported 81% LC at 1 year in a population with in-field failure following previous SBRT to spinal metastases. Sahgal et al. (71) reported no statistically significant difference in LC between those who had and had not received prior radiotherapy (*P* = .09, overall LC at 1 year = 85%). Similarly, Nikolajek et al. (72) reported local failure in 12.9% of previously irradiated patients; PR rates are not reported, only an improvement in median VAS post-treatment in patients with pain prior to SBRT (*P* = .002). From a PR perspective, both Mahadevan et al. (73) and Choi et al. (68) reported 65% TTP PR in a re-irradiation cohort (n = 34 and n = 23 patients, respectively). All of these studies included a mixed diagnostic cohort. Conversely, 10 of 45 LC studies and 11 of 38 PR studies excluded individuals who had received prior irradiation to the index site. TTP PR in these studies ranged from 47% to 92% (53, 74). LC ranged from 25% at 1 year (HCC, n = 63 lesions) to 96.5% (mixed diagnoses, n = 811 lesions) (24, 36), with a small study of seven patients reporting 100% LC in a mixed diagnostic population (47). In the remaining studies, the proportion who had undergone prior irradiation was either unclear or outcomes were not reported separately.

A small number of studies assessed the relationship between re-irradiation following cEBRT and PR or LC. Chang et al. (32) reported no statistically significant difference in PR or LC at 6 months, 1 year, or 2 years for patients who had or had not undergone previous cEBRT to the index lesion, although both outcomes were superior in those not previously irradiated (n = 54 re-irradiation, n = 131 initial treatment). LC at 1 year was 89.2% in patients undergoing initial treatment vs 80.8% in those undergoing re-irradiation (*P* = .093), whereas PR at 6 months, in assessable patients, was 92.9% and 86.4%, respectively (*P* = .314). Similarly, Laufer et al. (67) reported a statistically nonsignificant reduction in progression-free survival in patients who failed prior cEBRT (SHR = 1.96, 95% CI = 10.9 to 33.6, *P* = .07).

### Outcome Assessment

The measurement and definition of PR varied widely between studies. Seventeen PR (44.7%) studies reported no information about measurement tools or response definition. In those where this information was included, a majority used a 0–10 NRS or VAS for measurement of pain. Response definitions varied: four studies (10.5%) used the ICPRE (36, 38, 54, 63), and a further two used the Radiation Therapy Oncology Group 0631 trial protocol definitions (64, 75). These studies report TTP PR of 60%–81%. A further four studies reported adjusting response rates for analgesia (31, 42, 61, 73). TTP PR rates in these studies ranged from 65% (73) to 88% (61). The largest of these series (n = 336) reports long-term pain improvement in 86% of treated lesions (31). In this study, however, it is unclear how adjustment for analgesia was made. Long-term pain improvement was defined as pain control at last clinical review, although no protocol for follow-up frequency was reported. Unusually, Heron et al. (30) asked patients to classify their pain relative to baseline at 1–2 weeks and at intervals up to 12–24 months.

In 16 of 38 studies (42%), patient-reported outcomes were used (28, 31, 36–43, 49, 54, 57, 63, 76, 77). In the remaining studies, this was not clearly reported. Where patient-reported outcomes were reported, the mechanism of collection was frequently not clear. In addition, the time point used to report PR was unclear in a majority of studies (21 of 38).

A limited number of studies reported rates of attrition due to either death or loss to follow-up. As such, it was often unclear if outcomes were for assessable patients only or on a TTP basis. Where both are reported, the TTP PR result was lower than for assessable patients only [(62% vs 82% (55), 47% vs 62% (74), 60% vs 69% (38), 77% vs 91% (32), 61% vs 93% (68), and 71% vs 88% (4, 61)]. Overall response rates for PR varied from 38% (n = 28) (34) to 100% (n = 18) (52) in assessable patients.

Four studies reported the duration of response. Lee et al. (61) showed a median duration of pain relief of 3.2 months (range = 1–30 months) after SBRT in 57 patients with spinal metastases. A small study including 18 patients with bone metastases from RCC found that 32% of patients who responded had a symptomatic recurrence after a mean of 2.3 months (56). In two larger, mixed diagnosis studies, Hunter et al. (64) reported durability of PR of 4.8 months following SBRT, whereas Ryu et al. (42) reported a much longer median duration of PR of 13.6 months in 49 patients with a single isolated spinal metastasis. Notably, the frequency of follow-up in these studies was not clearly reported (56, 64), reported to be flexible (42), or stated as every 1–3 months (61).

LC was assessed based on a range of imaging modalities with computed tomography, magnetic resonance imaging, and positron emission tomography being most frequently used. Where LC was defined, it was stated to be an absence of radiological tumor growth indicating stable disease (19 of 45). Some studies also accounted for pseudoprogression (ie, when changes occur soon after SBRT that can mimic true progression) (23, 58, 70). Response Evaluation Criteria in Solid Tumours criteria were used in 4 of 45 studies (47, 50, 59, 72). McDonald et al. (59) made comparisons between these criteria and the MD Anderson Cancer Center criteria, demonstrating a discrepancy of up to 10% in LC rates depending on the criteria used for response assessment. As with the assessment of PR, limited information was reported about the time point for assessments, although many studies reported that scans took place at regular intervals (predominantly every 3 months) and used a time-to-event–based analysis.

Finally, in a majority of studies where both patient and lesion numbers were reported, a proportion of patients received treatment to more than one lesion (33 of 37 PR and 36 of 44 LC studies). It was often unclear if these outcomes were reported on a per patient or per lesion basis.

### Within Study Comparisons to Conventional Radiotherapy

Berwouts et al. (38) reported a small randomized phase II trial in which 12 patients received cEBRT with a dose of 8 Gy, 14 received 6.1–10 Gy dose-painting by numbers (DPBN), and 13 received a single 16 Gy fraction using DPBN. This technique aims to deliver higher doses to areas with an increased standardized uptake value on PET while de-escalating the dose to surrounding tissues. The latter two arms were delivered using an Intensity Modulated Radiation Therapy technique, and all anatomical sites were included. Overall response rates were 53%, 80%, and 60% at 1 month, respectively (38). Hunter et al. (64) compared PR in 110 patients with RCC treated with SBRT (n = 76) or cEBRT (8–30 Gy in 1–10 fractions, n = 34). They reported no difference in the proportion with PR (62% vs 68%, respectively), although cEBRT was associated with higher partial response (56% vs 29%) and lower complete response (12% vs 33%). A statistically non-significant increase in durability of PR was seen (1.7 vs 4.8 months, *P* = .095), but the difference in follow-up between the cohorts makes this difficult to interpret. No OS outcomes were reported, although KPS was statistically significantly higher (*P* < .001) in the SBRT group.

Gagnon et al. (52) reported no statistically significant difference in PR following SBRT and cEBRT in a matched cohort of 18 patients with breast cancer (prior cEBRT having been given to 17 SBRT patients). Patients were matched on interval from initial diagnosis, presence of visceral metastases, tumor differentiation, prior radiotherapy, and PS, among others. Similarly, Sohn et al. (36) reported outcomes for a matched cohort of 28 patients with HCC. The decrease in VAS was greater in the SBRT cohort than the cEBRT cohort (3.7 vs 2.8, *P* = .13) and the overall PR higher (64% vs 57%, *P* = .83), although neither of these differences were statistically significant. No difference was observed in median LC (*P* = .48). A similar analysis by the same group considered a cohort of 13 patients with RCC who received SBRT (54). The decrease in VAS was statistically significantly larger in the SBRT cohort (*P* = .04), although PR rates were again not statistically significantly higher (*P* = .63). Progression-free survival was statistically significantly higher in the SBRT cohort (*P* = .01).

Subsequent to the initial submission of the current study, a further, randomized phase II trial assessing the relative efficacy of a 24 Gy single SBRT fraction and 30 Gy in 10 fraction cEBRT was reported by Sprave et al (78). Given this is the highest level of evidence available currently, it is included here for information. This single institution study randomized 55 patients with painful spinal metastases from confirmed solid-organ malignancy, with good PS and no prior irradiation or neurological impairment. The authors report a more rapid improvement in pain following SBRT than cEBRT (*P* = .01) alongside higher rates of PR at 6 months (73.7% vs 35%, respectively, *P* = .015). These outcomes are in keeping with those reported by Hunter et al. (64): the rate of complete pain response rising (43.5% at 3 months following SBRT vs 17.4% following cEBRT) and the partial response rate falling (26.1% and 30.4%, respectively). Notably, the PR rates at 6 months post-cEBRT are relatively poor. Although encouraging for a possible benefit of SBRT relative to cEBRT, this is a small single-center study and is therefore not conclusive. Of the included patients, 11 (22%) reported pain scores of less than 2 at baseline; their distribution between the trial arms is unclear, introducing a risk of bias. In addition, the measurement of pain was non-standard both in terms of frequency (daily reports rather than worst pain over the preceding 3 days) and the use of a 0–100 VAS, making comparisons difficult.

### Toxicity Outcomes

Overall, 40 out of 57 studies reported on toxicity. The largest prospective study (a non-randomized phase I–II trial) analyzed toxicity in 149 patients using patient-reported outcomes and stated that toxicity was mostly mild, including grade 1–2 transient numbness and tingling, nausea, and vomiting (28). Grade 3 toxicities included pain, gastrointestinal disturbance, fatigue, and diaphoresis (overall 12 events, patient numbers unclear), although no radiation-related spinal cord myelopathy was reported (28). Similarly, Garg et al. (39) reported a total of 48 grade 1–2 toxicity events (patient numbers unclear), and 2 patients experienced grade 3–4 neurotoxicity. Berwouts et al. (38) reported that pain flare was observed in 25% of patients undergoing 16 Gy DPBN in their small randomized phase II study. Including these studies, overall, 10 studies, including 676 patients, recorded toxicity prospectively and reported grade 3 or 4 toxicity in 19 patients (0.03%) (28, 37, 40, 41, 45, 46, 49, 63, 68). All other grade 3–4 toxicities reported in prospective studies were treatment-related neurologic toxicity (39, 41, 46, 68). Of the 28 studies evaluating toxicity retrospectively, 5 grade 3 or 4 toxicities (0.002%) were observed in 2033 patients (22, 24, 25, 29, 30, 32, 34–36, 52–57, 59, 60, 66, 69–71, 73–77, 79, 80).

## Discussion

This systematic review assessed the available literature reporting the effectiveness of SBRT for bone metastases in patients with advanced cancer. A majority of studies report LC rates of more than 80%, with more than half reporting TTP PR rates above 75%. Reported toxicity was generally low grade, with high-grade toxicity rare overall. As such, SBRT appears to be a safe treatment for bone metastases with PR rates superior to those reported following cEBRT (TTP PR 61%) and excellent LC rates (3, 4, 81). There is, however, a risk of publication bias in this setting, with studies reporting superior outcomes potentially being preferentially published. In addition, given that the available literature represents predominantly non-randomized observational studies, we must be critical in interpreting these data because a number of aspects of the included studies may give rise to biased estimates of treatment effect.

The variable definition of PR used between studies is critical. The impact of cEBRT fractionation on PR in bone metastases was the focus of extensive international study. Thanks to these studies, the need to ensure comparable outcome reporting was recognized, leading to the collaborative development and publication of ICPRE for reporting outcomes (20, 82). It is remarkable that the majority of included studies did not adhere to these endpoints despite most being conducted after 2002. Of particular concern is the failure of many studies to adjust for changes in analgesic intake, retrospective designs, and the use of clinician-reported outcomes (65). A number of studies reported only average pain scores pre- and post-treatment rather than response rates. This limits comparability with existing cEBRT trials reporting response rates. It is also not possible to know to what extent the observed improvement reflects the effects of other interventions (eg, analgesia, systemic therapies) or indeed, regression to the mean. This latter may occur when patients with severe pain at baseline report improvements that simply reflect variation over time (83). Further, even in studies where SBRT was reportedly delivered for pain control, patients without pain at baseline were included (28, 41, 42, 66). Notably, those studies where response was assessed in line with ICPRE report lower PR rates (TTP PR 60%–77%), more in keeping with those seen in cEBRT and the limited randomized data now available (78).

In addition, in most studies it was unclear whether PR was reported only for assessable or for all treated patients; both are necessary in this fragile patient population where assessment of pain is complex and survival is limited. In studies carried out in palliative populations, missing data are inevitable. Complex methods to handle this are available (84), however, at a simple level, recognition and reporting of the extent and potential consequences of missing data is required (85). In many of the included studies, it was unclear to what extent data were missing. The consequences of this were rarely recognized and the limitations of the study outcome not discussed. Where both assessable and TTP results were reported, assessable PR was higher than TTP PR. As such, variable response definitions and reporting may explain some of the variation observed between studies, contributing to the relatively high rates of PR reported.

In both PR and LC studies, measurement time points during follow-up were frequently not well defined. Rather than response rates reflecting the proportion experiencing a response at, for example, 1 month or 3 months post-treatment, any response during follow-up was accepted. This may result in the outcomes being influenced by subsequent local or systemic treatments rather than SBRT. Conversely, in defining LC rates, the majority of studies used a Kaplan-Meier analysis. This analysis fails to acknowledge competing risks, assuming that those patients who die prior to assessment are censored and have an equivalent risk of recurrence to that of the surviving population—an unobservable assumption not supported by, for example, Bishop et al. (33, 86).

A lack of clarity exists over the optimum way to assess radiological response in bony lesions (59). Where reported, LC was usually defined as an absence of tumor volume change consistent with radiologic progression. As the radiotherapy community is gaining more experience with SBRT, it becomes clear that pseudoprogression and tissue necrosis are important factors to consider after SBRT (58, 87). Only four studies accounted for pseudoprogression by obtaining confirmatory scans before the lesion was classified as progressing, potentially resulting in an underestimation of LC. Notably, McDonald et al. demonstrated the impact that variable LC definitions can have on reported outcomes (59). Blinding was also not present in a majority of studies, potentially introducing bias in the assessment of LC. In addition, the value of LC as an outcome measure in palliative trials is questionable. If the accruing evidence for the use of SBRT in oligo-metastatic disease demonstrates improved survival outcomes, the value of LC as an endpoint might increase (88–91). In the absence of this confirmation, however, the assessment of clinically relevant outcomes of importance to patients should take priority: quality of life and pain control.

Finally, toxicity data were predominantly reported by clinicians and collected retrospectively, potentially leading to the under estimation of the rates seen. Overall, in the included prospective studies severe toxicity was rare (0.03% grade 3 or higher). Limited information was available to quantify low-grade toxicity. It is notable that rates of toxicity (both low and high grade) were markedly higher in the included early phase clinical trials than in observational cohorts (28, 38, 39). Given the wide discrepancies seen, further information is required, as even low-grade toxicity may still impact on quality of life and therefore remains important in informing clinical decisions.

In observational studies, bias may result from a number of methodological and reporting limitations (62, 63). As measurement error is discussed above, two other major causes of bias warrant discussion: selection bias and confounding. These result from variation between treatment cohorts in observational studies. If the predictors of treatment allocation or associated features (confounders) also influence outcomes, treatment effect estimates will be biased. Adjustment can be made for observed variation; however, unobserved variation persists. In this case, the variable inclusion, lack of separate outcome reporting, and lack of adjustment for baseline covariables may all contribute to the estimated effect of SBRT being biased.

The factor most likely to be having this affect is selection bias. It is notable that the median survival of patients included in these studies (8–47 months) was clinically significantly higher than that of randomly assigned individuals in previous cEBRT phase III trials (median 7 months) (92) or, indeed, in the routinely treated population (median, 4.8 months) (93). This may reflect exclusion of patients with limited follow-up or poor PS (37, 65). It has been previously demonstrated that patients with survival of more than 1 year following treatment have superior PR following an 8 Gy single fraction of cEBRT (85%–87%) compared to those surviving less than 3 months (44%–47%) (94, 95). Within the included studies, Garg et al. (39) demonstrate a positive correlation between PR and survival, whereas Berwouts et al. (38) report some of the lowest PR rates alongside the shortest survival time. In addition, Lee E et al. add to previous reports (Westhoff et al and van der Velden et al) demonstrating better PS is predictive of higher PR (61, 96, 97). Overall, the reported outcomes align more closely with those previously reported for individuals with favorable prognosis. It is clear from the reported survival times that the populations included in a majority of these studies are highly selected, with higher than average survival, better PS, and favorable diagnostic case mix. Selection bias is, thus, a major limitation of the included non-randomized studies (64).

Other sources of heterogeneity in the treated populations that may potentially impact the reported outcomes include the variable inclusion of patients with MSCC and prior irradiation. In contrast to the possible impact of prolonged survival on response rates, these factors may result in reduced response rates as compared to previously published cEBRT studies in bone metastases. For example, Lee S et al. (61) demonstrated that the presence of MSCC predicts for a lower probability of pain response (*P* = .001) on multivariable modeling, whereas prior irradiation was reported to be associated with a statistically nonsignificant reduction in PR (25, 67).

Finally, heterogeneity is present in the considered studies as a result of varying treatment regimens. This may result from variation and evolution in the dose-fractionation schedules used, incorporation of surgery into the treatment pathway, or concurrent and subsequent systemic therapy.

The heterogeneity resulting from the above outlined study variation is clinically significant and likely to result in biased estimates of treatment effect. For example, previous trials of dose fractionation in palliative radiotherapy focused exclusively on bone metastases or, separately, MSCC and excluded patients undergoing prior radiotherapy or surgery to the index lesion. Ideally, a meta-regression approach may allow investigation of how these factors impact outcomes; however, the quality of reporting in the included studies did not support this (98).

It is clear from the above discussion that the outcomes reported in many of the included studies are not directly comparable to those that might be expected in a routine patient population, including those with bone metastases beyond the spine. Indeed, where attempts have been made to provide a comparator cohort, either through randomization of a small number of patients or through an observational matched cohort design, the differences in PR are markedly less than might be anticipated based on the single-arm series. Given the clear challenges of selection bias in observational studies, risk of measurement bias and marked heterogeneity in the studies included here, further large randomized trials are required to assess the role of SBRT in the management of bone metastases (99–101).

In 2002 ICPRE were published to guide the conduct and reporting of randomized studies in this area, with subsequent updates a decade later (1, 65). Specifically, they provided definitions of pain response alongside recommendations for how and when to measure pain response. Despite this, a minority of studies incorporated these recommendations (10, 25, 27, 28), with a further limited number using alternative methods based on published randomized trial protocols (29–31). The emphasis of trials comparing SBRT with cEBRT is likely to be on the potential for improved quality and durability of pain control, yet, very few of the studies included here have attempted to address these endpoints. A limited number of studies recognized and robustly reported complete and partial PR separately. Where durability has been considered, it has been measured by “long-term pain control” or as a component of a composite progression-free survival endpoint (31, 53). These outcomes are not well defined, and it is unclear if the methods used reflect the hoped-for outcome: a greater proportion of remaining life spent with pain control. Net pain relief measures this outcome. It has, however, been reported in a limited number of studies, is not strongly recommended in the ICPRE, and has limited information available to support its value as an outcome measure (6, 20, 102).

It may be argued, therefore, that the ICPRE are not well suited to assessing the role of SBRT for bone metastases. To support future trials, the existing endpoints should be reviewed. The update and implementation of such endpoints will help ensure that the patient group, intervention, and comparator treatments are clearly specified and that the outcomes measured are defined and measured robustly, reflecting the question to be addressed and critically offering value to patients.

In conclusion, the studies included in this systematic review report higher rates of pain response following SBRT than have previously been reported following cEBRT. Local control is excellent with limited toxicity. However, these outcomes may very well be the result of study methodology and, most importantly, selection bias. Early randomized trial outcomes are encouraging but urgently require replication in larger studies. In addition, there is a need to update the ICPRE to better support the investigation of the role of SBRT in bone metastasis management, specifically focusing on response durability. This systematic review supports recruitment into existing randomized trials of SBRT for bone metastases and illustrates the need for further trials in this setting.

## Funding

This work was supported by the Medical Research Council UK (grant number MR/N021339/1 to KLS).

## Notes

Affiliations of authors: Cancer Epidemiology Group, University of Leeds, Leeds, UK (KLS); Departments of Radiation Oncology and Orthopedic Surgery (JMvdV, ES, JJV, HMV), and Julius Center for Health Sciences and Primary Care (HMV), University Medical Center Utrecht, Utrecht, The Netherlands; Sunnybrook Odette Cancer Centre, University of Toronto, Toronto, ON, Canada (EW, AS, EC); Department of Radiotherapy, Leiden University Medical Center, Leiden, The Netherlands (YMvdL).

The funder had no role in the design of the study; the collection, analysis, and interpretation of the data; the writing of the manuscript; and the decision to submit the manuscript for publication.

AS reports advisory and/or consultant roles with AbbVie, Merck, Roche and Varian (Medical Advisory Group), Elekta (Gamma Knife Icon); ex officio board member of International Stereotactic Radiosurgery Society; past educational seminars with Elekta AB, Accuray Inc, Varian (CNS Teaching Faculty), BrainLAB, Medtronic Kyphon; research grant with Elekta AB; travel accommodations and/or expenses by Elekta, Varian, BrainLAB. AS also belongs to the Elekta MR Linac Research Consortium, Elekta Spine, Oligometastases, and Linac Based SRS Consortia. All other authors have no conflicts of interest directly related to this work.

We are grateful to a number of authors for providing additional data to support this analysis. We furthermore thank the librarian team at Utrecht University for their assistance in developing the search strategy.

## Supplementary Material

djz101_Supplementary_Data
