# Supplementary material for: Systematic Review of the Role of Stereotactic Radiotherapy for Bone Metastases
Source: J Natl Cancer Inst. 2019 May 23;111(10):1023–32. doi: 10.1093/jnci/djz101 (PMC6792073; doi:10.1093/jnci/djz101)
Supplement: djz101_Supplementary_Data [file djz101_supplementary_data.pdf]

SUPPLEMENTARY MATERIALS

Supplementary Methods

In conducting the systematic review the following search terms were used:

"bone and bones" OR bone OR bones OR bony OR skeletal OR osseous OR spine OR spinal

AND

neoplasmata OR metastasis OR metastases OR metastatic OR neoplasm OR neoplasms OR cancer OR cancers OR carcinoma OR carcinomas OR tumor OR tumors OR tumour OR tumours

AND

radiosurgery OR "stereotactic body radiotherapy" OR "stereotactic body radiation therapy" OR "stereotactic body radiosurgery" OR "stereotactic radiosurgery" OR "stereotactic spinal radiotherapy" OR "stereotactic spinal radiosurgery" OR stereotaxis OR sbrt OR srs OR sbrs OR ssr OR sabr OR "stereotactic ablative"

Supplementary Tables

Supplementary Table 1. Summary of Findings for studies reporting on pain relief after SBRT.

Reference	Enrolment period	Study type	Sample size (lesion/ patients)	Study population	Primary tumor	Location	Dose, Fractions	Outcome definition	Outcome
Amini et al. 2016* ¶ (1)	2004-2014	RC	50 lesions	NR	RCC	Mixed	Most common 27 Gy in 3 fractions	Clinical symptoms based on patient report and Wong-Baker Faced pain rating Scale when recorded	Complete response 36.8%; Partial response 44.7%; stable disease 5.3%; and progressive disease 13.2%
Anand et al. 2015 ¶ (2)	2010-2012	RC	76 lesions in 52 patients	Patients with and without a prior history of surgery or radiotherapy	Mixed	Spine	14-27 Gy in 1-3 fractions	CR if pain disappeared completely, PR >50% relief on VAS score	Complete response 92.3% and Partial response 5.8% within 3-5 days
Azad et al. 2016 ¶ (3)	2005-2013	RC	25 lesions in 25 patients.	Patients without a prior history of any treatment to the index site	Mixed	Craniove-rtebral junction	15-25.5 Gy in 1-5 fractions	NR	17 patients had pain at baseline. Marked decrease or full resolution of pain in 8 patients, no change in 5 patients, 4 patient unknown
Bate et al. 2015 ¶ (4)	2007-2011	RC	69 lesions in 57 patients	Patients with or without a prior history of separation surgery	Mixed	Spine	16-30 Gy in 1-5 fractions	NR	Data on pain were available for 45 cases, with an overall VAS score decrease of 3.4 ±2.6 following treatment
Berwouts et al. 2015 (5)	2010-2014	Phase II RCT‡	15 patients	Patients without a prior history of radiotherapy or treatment with radionuclides	Mixed	Mixed	16 Gy in 1 fraction	According to the ICPRE(6)	Overall response of 60%, overall response 69% in assessable patients only
Chang et al. 2012 ¶ (7)	2002-2008	RC	185 lesions in 142 patients	Patients with or without a prior history of radiotherapy to the index site	Mixed	Spine	17.9-23.7 Gy	NR	Pain control rate at 6 months was 86% in the retreatment group, and 93% at the initial treatment group

Choi et al. 2010 ¶ (8)	2002-2008	RC	51 lesions in 42 patients	Patients with a history of prior radiotherapy to the index site	Mixed	Spine	10-30 Gy in 1-5 fractions	NR	65% reported significant pain relief, outcome not available in 8 patients, 1 patient without pain relief
Gagnon et al. 2007 (9)	2002-2005	RC‡	18 patients	Women who failed prior conventional radiotherapy	Breast cancer	Spine	21-24 Gy in 3 fractions	NR	Pain relief within the treated site was judged near-complete in all patients
Garg et al. 2012‡ (10)	2005-2010	Ph I/II	63 lesions in 61 patients	Patients with previously unirradiated lesions	Mixed	Spine	18-24 Gy in 1 fraction	NR	More patients experienced reduced pain levels
Germano et al. 2016 ¶ (11)	2007-2014	RC	143 lesions in 95 patients	Patients with or without a prior history of surgery and/or radiotherapy	Mixed	Spine	10-18 Gy in 1 fraction	Improvement if at least 2 points decrease on VAS	Pain improvement resulted within 7 days in 100% of the patients with severe pain
Gerszten et al. 2005 (12)	NR	PC	26 lesions in 26 patients	Patients undergoing kyphoplasty-based closed fracture reduction	Mixed	Spine	16-20 Gy in 1 fraction	NR	Long-term improvement in back pain occurred in 24 of 26 patients (92%)
Gerszten et al. 2007 ¶ (13)	NR	PC	336 lesions treated for pain	Patients with or without a prior history of surgery and/or radiotherapy	Mixed	Spine	12.5-25Gy in 1 fraction	Pain score improvement of at least 3 points on VAS score	Overall long-term improvement of in pain in 290 of the 336 cases (86%)
Gibbs et al. 2007 (14)	1996-2005	PC	102 lesion in 74 patients	Patients with or without prior treatment to the index site	Mixed	Spine	16-25 Gy in 1 fraction	NR	In 62 patients with pain and/or neurological dysfunction, 52 patients (82%) reported improvement or resolution of symptoms
Heron et al. 2012§¶ (15)	2000-2008	RC	153 lesion in 104 patients	Patients with or without a history of prior irradiation	Mixed	Spine	20.6-Gy 24.5 in 3-5 fractions	Long-term decrease in pain measured after 4-6 months	73% in multiple fraction group experienced pain relief
Hsu et al. 2015† (16)	2007-2013	RC	32 lesions in 32 patients	Patients with or without prior surgery	Mixed	Spine	7.6-50 Gy in 1-5 fractions	NR	Mean VAS before SBRT was 8.8 and

									decreased to 2.5 after 1 month
Hunter et al. 2012* (17)	2002-2010	RC	76 patients	Patients with or without a history of prior radiotherapy	Mixed	Spine	8-16 Gy in 1 fraction	Scored according to the RTOG 0631 protocol (18)	Overall pain response 62%, Complete response 33%; Partial response in 29%
Hwang 2012 ¶ (19)	2003-2010	PC	11 lesions in 8 patients	Patients with osteoblastic lesions without a prior history of surgery or radiotherapy to the index site	Mixed	Spine	Average 34.5 Gy in 1-6 fractions	At least 2 points decrease after 1 month	All patients had at least 2 points decrease, no patients with pain score of 0
Jhaveri et al. 2012 (20)	2004-2006	RC	24 lesions in 18 patients	Patients with no history of prior surgery or radiotherapy to the index site	RCC	Mixed	18-40 Gy in 3-4 fractions	Patient reported decrease in pain score on VAS at follow-up visits	14 out of 18 patients (78%) had a pain response
Kim et al. 2013 (21)	2009-2010	PC	31 lesions in 22 patients	Patients with or without a history of prior surgery or irradiation	Mixed	Spine	16-30 Gy in 1-5 fractions	According to ICPRE (6)	Complete follow-up at 3 months, CR 52%, PR 19%, SD 26%, PD 3%
Ksiezniak-Baran et al. 2015 ¶ (22)	NR	RC	33 lesions in 28 patients	(Oligo)metastatic patients with our without a history of prior surgery	Mixed	Spine	8-40 Gy in 1-3 fractions	NR	11 patient with pain, stable in 5 patients, 3 improved.
Lee et al. 2012 (23)	2007-2009	RC	73 lesions in 57 patients	Patients with or without a history of prior surgery or irradiation	Mixed	Spine	15-35 Gy in 1-5 fractions	Decrease of at least 3 points on VAS score without increase in analgesic use, CR if no analgesics or VAS 0-1	Pain relief was achieved in 59 out of 67 painful lesions (88.1%); Complete response in 34 lesions (51%)
Lee et al. 2013 ¶ (24)	2009-2011	RC	51 lesions in 36 patients	Patients with or without a history of prior irradiation	Mixed	Spine	12-36 Gy in 1-6 fractions	NR	Pain relief was assessable in 29 lesions; 72.4% achieved a response within 1 week
Lee E et al. 2015‖¶ (25)	2008-2012	RC	15 lesions in 13 patients	Patients without a prior history of surgery or radiotherapy with no concurrent systemic treatment	HCC	Spine	18-40 Gy in 1-4 fractions	Pain response if a pain score at follow-up was lower than baseline	Of 13 assessable lesions at 3 months, 12 lesions showed improvement

Lee SH et al. 2015 ¶ (26)	2010-2014	RC	63 lesions in 47 patients	Patients without a history of prior surgery	Mixed	Spine	26-42 Gy in 4-6 fractions	Scored according to the RTOG 0631 protocol (18)	From 46 assessable patients, 10 (21.7%) had CR and 28 (60.9%) patients had PR
Mahadevan et al. 2011 ¶ (27)	2005-2008	RC	81 lesions in 60 patients	Patients with radiological and/or clinical progression after prior external beam RT	Mixed	Spine	24-30 Gy in 3-5 fractions	NR	1 month after reirradiation, 22 out of 34 patients with pain had improvement (64.7%)
Massicotte et al. 2012 ¶ (28)	2009-2010	PC	10 lesions in 10 patients	Patients treated with minimal access spine surgery followed by SBRT	Mixed	Spine	18-35 Gy in 1-5 fractions	NR	8 patients with back pain; at 1 months post-treatment, median improvement 1 point on VAS, after 5 months median improvement 6 points on VAS
Muacevic et al. 2014 ¶ (29)	2005–2009	PC	64 lesions in 40 patients	Highly selected good prognosis subgroup harbouring 1 – 2 metastases, including postoperative or re-irradiation	Prostate	Mixed	16.5-22 Gy in 1 fraction	Pain status was defined by VAS	Pain reduction could be documented in 5 out of 6 patients with initial pain (83%).
Napieralski et al. 2016 ¶ (30)	2011-2015	RC	71 lesions in 51 patients	Oligometastases or oligo-recurrences	Prostate	Mixed	6-45 Gy in 1-5 fractions	Pain level	At last control, 32 patients with CR, 15 patients with PR, 4 patients with pain (92%).
Owen et al. 2014 ¶ (31)	2008-2012	PC	85 lesions in 74 patients	Patients with or without a history of prior irradiation	Mixed	Non-spine bone metastases (including sacrum)	15-50 Gy in 1-5 fractions	NR	36 patients with painful lesions, 88% experienced subjective improvement
Park et al. 2015 ¶ (32)	2008-2012	RC	59 lesions in 39 patients	Patients with or without a history of prior surgery or irradiation	Mixed	Spine	18-35 Gy in 1-5 fractions	NR	Median pre-SBRT VAS was 4 (range, 0-10), at 1-3 months after SBRT, median VAS of 1 (range, 0-8)

Ryu et al. 2008 (33)	2001-2003	PC	61 lesions in 49 patients	Patients with a single isolated spinal metastasis	Mixed	Spine	10-16 Gy in 1 fraction	CR no pain at 8 weeks without analgesics, PR reduction of pain score of at least 2, PD any increase in pain score or analgesics	Complete response at 8 weeks of 46%; Partial response 18.9%; and stable disease in 16.2%
Schipani et al. 2012 ¶ (34)	2005-2008	RC	165 lesions in 124 patients	Patients with 1 or 2 contiguous spine metastases	Mixed	Spine	18 Gy in 1 fraction	NR	114 patients (92%) had improvement in pain and/or neurological symptoms
Sheehan et al. 2009 ¶ (35)	NR	RC	110 lesions in 40 patients	Patients with or without a history of prior spinal surgery	Mixed	Spine	10-24 Gy in 1-5 fractions	Pain improvement	Improvement was seen in 34 patients (85%) Note; only 32 patients had pain at baseline
Sohn et al. 2014*¶ (36)	2005-2012	RC	At least 31 lesions in 13 patients	Patients without a history of prior treatment	RCC	Spine	Mean dose of 38 Gy in 1-5 fractions	According to ICPRE (6)	At 1 months, CR in 3 patients (23.1%) and PR in 7 patients (53.8%)
Sohn et al. 2016*¶ (37)	2005-2012	RC	At least 63 lesions in 28 patients	Patients without a history of prior treatment	HCC	Spine	Mean dose of 36 Gy in 1-5 fractions	According to ICPRE (6)	At 1 months, CR in 6 patients (21.4%) and PR in 12 patients (42.9%)
Staehler et al. 2010†¶ (38)	2005-2009	RC	105 lesions in 55 patients	Patients with progressive disease with a life expectancy of at least 3 months	RCC	Spine	20 Gy in 1 fraction	NR	Median VAS decreased from 5 to 0 within 1 week, only 2 lesions not controlled after 6 months
Tsai et al. 2009 ¶ (39)	2005-2007	RC	127 lesions in 69 patients	21% patients received previous radiotherapy	Mixed	Spine	10-30 Gy in 1-5 fractions	NR	Overall VAS improvement was found in 110 lesions
Wang et al. 2012 (40)	2002-2011	Ph I/II	166 lesions in 149 patients	Oligometastatic patients, or failure after prior surgery or radiotherapy	Mixed	(Para)spinal lesions	27-30 Gy in typically 3 fractions	Frequency of complete pain relief	After 6 months, 55 of 120 patients (45.8%) experienced CR

Abbreviations: CR, complete response; CT, computed tomography scan; Gy, Gray; HCC, hepatocellular carcinoma; ICPRE, International consensus on palliative radiotherapy endpoints; MRI, magnetic resonance imaging; MSCC = malignant spinal cord compression; NR, not reported; NRS, numeric rating score; PC, prospective cohort study; PD, progressive disease; PET, positron emission tomography; PFS, progression free survival; PR, partial response; RC, retrospective cohort study; RCC, renal cell carcinoma;

RCT, randomized controlled trial; RT, radiotherapy; SBRT, stereotactic body radiotherapy; SD, stable disease; TKI = tyrosine kinase inhibitors; VAS, visual analog scale.

*Only results for the SBRT group are included in this Table.
†Only results for patients with bone metastases are included in this Table.
‡Results on local control are reported in Bishop et al 2015.
§Only the results for the multiple fractions group is included, the results for the single fraction group were reported in Gerszten et al 2007.
||Only the results for patients treated with 1–4 fractions are included in this Table; 12 patients treated with 10 fractions were excluded.
¶ These studies report outcomes for pain response as well as for local control (see Table 2).

Table 2. Summary of Findings for studies reporting on local control after SBRT.

Reference	Enrolment period	Study type	Sample size (lesion/ patients)	Study population	Primary tumor	Location	Dose, Fractions	Outcome definition of local control	Outcome
Ahmed et al. 2012 (41)	2008-2010	PC	85 lesions in 66 patients	Patients with and without a prior history of radiotherapy and surgery	Mixed	Spine	10-40 Gy in 1-5 fractions	No progressive tumor growth on MRI or PET	89.2% at 1 year
Al-Omair et al. 2013 (42)	2008-2012	PC	80 lesions in 80 patients	Patients who were operated and treated with postoperative SBRT	Mixed	Spine	18-40 Gy in 1-5 fractions	No progression based on MRI	84% at 1 year, time to local failure 19.9 months
Amini et al. 2016*¶ (1)	2004-2014	RC	50 lesions	NR	RCC	Mixed	Most common 27 Gy in 3 fractions	No evidence of disease or decrease based on PET, MRI or CT	82.5%, 74.1% and 61.4% at 10, 12 and 24 months
Anand et al. 2015 ¶ (2)	2010-2012	RC	76 lesions in 52 patients	Patients with and without a prior history of surgery or radiotherapy	Mixed	Spine	14-27 Gy in 1-3 fractions	Either regression or non-progressive radiological response	Overall response 86.1% with a median follow-up of 8.5 months
Azad et al. 2016 ¶ (3)	2005-2013	RC	25 lesions in 25 patients	Patients without a prior history of any treatment to the index site	Mixed	Craniove-rtebral junction	15-25.5 Gy in 1-5 fractions	Radiographically determined	Lesion size decreased or unchanged in 16 patients, increased in 3 patients, 6 patients unknown
Bahig et al. 2016 (43)	2009-2014	RC	49 lesions in 35 patients	Patients with or without a prior history of surgery	Mixed	Spine	16-35 Gy in 1-5 fractions	Recurrence was defined as continuous tumor volume enlargement on >2 serial MRI studies over a period of >6 months and/or histologic confirmation	14 spinal segments developed local recurrence at a median time of 15 months, 1-year local control was 87%
Bate et al. 2015 (4)	2007-2011	RC	69 lesions in 57 patients	Patients with or without a prior history of separation surgery	Mixed	Spine	16-30 Gy in 1-5 fractions	Regression or stability of local tumor volume on MRI	94.2% local control at 1 year, 6 patients with local failure

Bishop et al. 2015 (44)	2002-20123	PC	332 lesions in 285 patients	Patients without a prior history of surgery	Mixed	Spine	18-27 Gy in 1-3 fractions	Absence of recurrence, recurrence defined as radiographical progression on MRI	Actuarial 1- and 3-year rates of local control were 88% and 82%, respectively
Chang et al. 2012 ¶ (7)	2002-2008	RC	185 lesions in 142 patients	Patients with or without a prior history of radiotherapy	Mixed	Spine	17.9-23.7 Gy	No evidence of mass regrowth on MRI or PET-CT	At 1 year, in the retreatment group 81% and 89% in the initial treatment group
Chang et al. 2017 (45)	2010-2014	RC	72 lesions in 60 patients	Oligometastatic patients with spinal metastases (up to 3 lesions)	Mixed	Spine	16-52.5 Gy in 1-3 fractions	NR	1-year local progression free survival was 85%
Choi et al. 2010 ¶ (8)	2002-2008	RC	51 lesions in 42 patients	Patients with a history or prior radiotherapy	Mixed	Spine	10-30 Gy in 1-5 fractions	Lack of progression within the treated vertebral body on MRI	73% at 1 year
Colaco et al. 2016 (46)	2008-2014	RC	86 lesions with 78 patients	Patients with spinal metastases and concurrent brain metastases	Mixed	Spine	10-27 Gy in 1-3 fractions	Absence of radiological changes suspicious of recurrence of disease on follow-up imaging	1-year local control of 89.4% with a median follow-up of 6 months
Deodato et al. 2013† (47)	NR	Ph I	8 lesions in 7 patients	NR	Mixed	NR	12-16 Gy in 1 fraction	No progression on CT or MRI according to RECIST criteria, or no increase in metabolic activity on PET	No local failures, 100% overall local control
Germano et al. 2016 ¶ (11)	2007-2014	RC	143 lesion in 95 patients	Patients with or without a prior history of surgery and/or radiotherapy	Mixed	Spine	10-18 Gy in 1 fraction	NR	Radiographic control (MRI) was in 94% (116/124) of cases
Gerszten et al. 2007 ¶ (13)	NR	PC	500 lesions in 393 patients	Patients with or without a prior history of surgery and/or radiotherapy	Mixed	Spine	12.5-25Gy in 1 fraction	NR	Long-term radiographic control was 90% when used as primary treatment, and 88% when used as ‘salvage’ technique
Gill et al. 2012 (48)	2005-2010	RC	20 lesions in 20 patients	Patients with oligometastatic disease with or without prior	Mixed	Spine	30-35 Gy in 5 fractions	No progression of the treated tumor on MRI and/or PET	1- and 2 year local control estimates are 80% and 73% respectively

				surgery to the index site					
Hamilton et al. 1996† (49)	NR	PC	8 patients	Patients with or without a history of prior irradiation	Mixed	Spine	8-10 Gy in 1 fraction	NR	No progression in all patients
Heron et al. 2012‡¶ (15)	2000-2008	RC	153 lesion in 104 patients	Patients with or without a history of prior irradiation	Mixed	Spine	20.6-24.5 Gy in 3-5 fractions	Tumor growth less than 25% was classified as local control	Long-term local control in 89%, at 2 years local control probability of 96%
Hwang 2012 ¶ (19)	2003-2010	RC	11 lesions in 8 patients	Patients with osteoblastic lesions without a prior history of surgery or radiotherapy to the index site	Mixed	Spine	Average 34.5 Gy in 1-6 fractions	Tumor growth less than 10% was regarded local control	10 out of 11 lesions locally controlled
Jahanshahi et al. 2012 (50)	2002-2008	RC	19 lesions	Patients with or without a prior history of surgery	Melanoma	Spine	NR	No local tumor progression	4 lesions CR, 4 stable, 11 unknown
Ksiezniak-Baran et al. 2015 ¶ (22)	NR	RC	33 lesions in 28 patients	(Oligo)metastatic patients with our without prior surgery	Mixed	Spine	8-40 Gy in 1-3 fractions	NR	In 17 patients, imaging available, 2 patients with progression
Laufer et al. 2013 (51)	2002-2011	RC	186 lesions in 186 patients	Patients with a history of prior surgery, with or without a history of prior radiotherapy	Mixed	Spine	24-30 Gy in 1-6 fractions	NR	Local control at 1 year 83.6%. Local progression in 34 patients
Lee et al. 2013 ¶ (24)	2009-2011	RC	51 lesions in 36 patients	Patients with or without a history of prior irradiation	Mixed	Spine	12-36 Gy in 1-6 fractions	Absence of disease progression on follow-up images	Local control at 1 year 88.2%, 6 cases of local failure
Lee E et al. 2015 ¶ (25)	2008-2012	RC	15 lesions in 13 patients	Patients without a prior history of surgery or radiotherapy with no concurrent systemic treatment	HCC	Spine	18-40 Gy in 1-4 fractions	Evaluated according to RECIST v1.1 and PERCIST v1.0, CR and PR were regarded radiological response	At 3 months, all lesions showed CR (n=7), PR (n=5) or SD (n=3), 1 year local control rate 78.6%
Lee SH et al. 2015 ¶ (26)	2010-2014	RC	63 lesions in 47 patients	Patients without a history of prior surgery	Mixed	Spine	26-42 Gy in 4-6 fractions	Absence of local tumor growth on MRI	Out of 27 assessable patients, local failure occurred in 4 lesions

Mahadevan et al. 2011 ¶ (27)	2005-2008	RC	81 lesions in 60 patients	Patients with radiological and/or clinical progression after prior external beam radiotherapy	Mixed	Spine	24-30 Gy in 3-5 fractions	NR	At last follow-up, 56 out of 60 patients had improved or stable disease in their scans
Massicotte et al. 2012 ¶ (28)	2009-2010	PC	10 lesions in 10 patients	Patients treated with minimal access spine surgery followed by SBRT	Mixed	Spine	18-35 Gy in 1-5 fractions	NR	3 patients had disease progression
McDonald et al. 2015 (52)	2011-2014	RC	42 lesions in 33 patients	Oligometastatic or oligo-progressive patients	Mixed	Non-spine bone	20-50 Gy in 1-5 fractions	Lesions that were classified as SD, PR, or CR according to RESIST v1.1 or MDA criteria	Overall local control according to RECIST of 36 out of 42 lesions ((86%), and 35 out of 42 lesions (83%) according to MDA criteria
Miller et al. 2016 (53)	2006-2015	RC	151 lesions in 100 patients	Patients with spinal metastases with or without concurrent treatment with TKIs	RCC	Spine	10-24 Gy in 1-3 fractions	Any in-field progression as evaluated by neuro-radiologists were considered local failure	At 12 months, local failure was lowest among patients treated concurrently with first-line TKI (4%) as compared to subsequent lines (27%).
Muacevic et al. 2014 ¶ (29)	2005–2009	PC	64 lesions in 40 patients	Highly selected good prognosis subgroup harbouring 1 – 2 metastases, including postoperative or re-irradiation	Prostate	Mixed	16.5-22 Gy in 1 fraction	Local failure if tumor growth on MRI or increased tracer uptake in choline PET-CT	Actuarial 6-, 12- and 24 months local tumor control rate was 95.5%
Napieralska et al. 2016 ¶ (30)	2011-2015	RC	71 lesions in 51 patients	Oligo-metastases or oligo-recurrences	Prostate	Mixed	6-45 Gy in 1-5 fractions (only 1 patient 6 Gy, 1 patient 8 Gy)	Lack of in-field progression	Follow-up available in 47 patients with 15 lesions in 10 patients progressed, 1 year local control rate 70%
Nikolajek et al. 2011† (54)	2005-2009	PC	41 patients	Patients with prior irradiation	Mixed	Spine	10-28 Gy in 1 fraction	Using RESIST criteria, local control defined as CR or PR	6 patients with a local failure

Owen et al. 2014 ¶ (31)	2008-2012	PC	85 lesions in 74 patients	Patients with or without a history of prior irradiation	Mixed	Non-spine bone metas-tases	15-50 Gy in 1-5 fractions	Stable disease, PR or CR based on serial imaging with CT, MRI, or PET	7 patients with in-field recurrence, 1 year local control rate of 91.8%
Park et al. 2015 ¶ (32)	2008-2012	RC	59 lesions in 39 patients	Patients with or without a history of prior surgery or irradiation	Mixed	Spine	18-35 Gy in 1-5 fractions	No tumor progression on imaging, or no salvage treatment	4 patients experienced local recurrence, 1 year local control rate 93.2%
Sahgal et al. 2009 ¶ (55)	2003-2006	RC	60 lesions in 37 patients	Patients with or without a prior history of surgery or irradiation	Mixed	Spine	7-40 Gy in 1-5 fractions	No progression based on imaging and/or symptoms	8 of 60 tumors recurred, 1 year PFS 85%
Schipani et al. 2012 ¶ (34)	2005-2008	RC	165 lesions in 124 patients	Patients with 1 or 2 contiguous spine metastases	Mixed	Spine	18 Gy in 1 fraction	NR	Local control in 114 patients (92%)
Sheehan et al. 2009 ¶ (35)	NR	RC	110 lesions in 40 patients	Patients with or without prior spinal surgery	Mixed	Spine	10-24 Gy in 1-5 fractions	Volume increase on MRI	Increase in volume in 20 lesions (18%)
Sohn et al. 2014*¶ (36)	2005-2012	RC	At least 31 lesions in 13 patients	Patients without a history of prior treatment	RCC	Spine	Mean dose of 38 Gy in 1-5 fractions	NR	Local control rate at 1 month of 100%, at 1 year of 85.7%
Sohn et al. 2016*¶ (37)	2005-2012	RC	At least 63 lesions in 28 patients	Patients without a history of prior treatment	HCC	Spine	Mean dose of 36 Gy in 1-5 fractions	NR	Local control rate at 1 month of 92%, at 1 year of 25%
Staehler et al. 2010†¶ (38)	2005-2009	RC	105 lesions in 55 patients	Patients with progressive disease with a life expectancy of at least 3 months	RCC	Spine	20 Gy in 1 fraction	NR	Local control rate after 12 months 94.1%
Thibault et al. 2014 (56)	2007-2012	PC	71 spinal segments in 37 patients	Patients with or without a history of surgery or irradiation	RCC	Spine	18-30 Gy in 1-5 fractions	Local progression based on radiologist's interpretation of MR images	Local progression in 12 of 71 spinal segments
Thibault et al. 2015 (57)	2009-2013	PC	56 lesions in 40 patients	Patients initially treated with SBRT who subsequently experienced local tumor progression	Mixed	Spine	20-35 Gy in 2-5 fractions	Local failure was defined as any tumor volume change consistent with radiologic progression	13 of 56 segments (23%) progressed locally, 12-months local control rate 80.6%

Tsai et al. 2009 ¶ (39)	2005-2007	RC	127 lesions in 69 patients	21% patients received prior radiotherapy	Mixed	Spine	10-30 Gy in 1-5 fractions	NR	Local failures were observed in 3 patients, 10-months local control was 96.8%
Ursino et al. 2016 (58)	2010-2013	RC	40 lesions in 40 patients	Oligometastatic patients without a history of surgery or irradiation	Mixed	Mixed	24 Gy in 1 fraction or 27 Gy in 3 fractions	NR	5 of 40 patients experienced a recurrence (12.5%)
Yamada et al. 2017 (59)	2003-2015	RC	881 lesions in 657 patients	Patients without a history of prior surgery or radiotherapy	Mixed	Spine	16-26 Gy in 1 fraction	No enlargement of the treated tumor on imaging studies or positive pathological findings after treatment	28 (3.5%) lesions progressed, with a 24-months local failure rate of 3.1%

*Only results for the SBRT group are included in this Table.
†Only results for patients with bone metastases are included in this Table.
‡Only the results for the multiple fractions group is included, the results for the single fraction group were reported in Gerszten et al 2007.
¶ These studies report outcomes for pain response as well as for local control (see Table 1).

References

1. Amini A, Altoos B, Bourlon MT, Bedrick E, Bhatia S, Kessler ER, et al. Local control rates of metastatic renal cell carcinoma to the bone using stereotactic body radiation therapy: Is RCC truly radioresistant?′. Pract Radiat Oncol. 2015;5(6):e589–96.

2. Anand AK, Venkadamanickam G, Punnakal AU, Walia BS, Kumar A, Bansal AK, et al. Hypofractionated Stereotactic Body Radiotherapy in Spinal Metastasis — With or Without Epidural Extension. Clin Oncol. 2015 Jun;27(6):345–52.

3. Azad TD, Esparza R, Chaudhary N, Chang SD. Stereotactic radiosurgery for metastasis to the craniovertebral junction preserves spine stability and offers symptomatic relief. J Neurosurg Spine. 2016 Feb;24(2):241–7.

4. Bate BG, Khan NR, Kimball BY, Gabrick K, Weaver J. Stereotactic radiosurgery for spinal metastases with or without separation surgery. J Neurosurg Spine. 2015 Apr;22(4):409–15.

5. Berwouts D, De Wolf K, Lambert B, Bultijnck R, De Neve W, De Lobel L, et al. Biological 18[F]-FDG-PET image-guided dose painting by numbers for painful uncomplicated bone metastases: A 3-arm randomized phase II trial. Radiother Oncol. 2015 May;115(2):272–8.

6. Chow E, Hoskin P, Mitera G, Zeng L, Lutz S, Roos D, et al. Update of the International Consensus on Palliative Radiotherapy Endpoints for Future Clinical Trials in Bone Metastases. Int J Radiat Oncol. 2012 Apr;82(5):1730–7.

7. Chang U-K, Cho W-I, Kim M-S, Cho CK, Lee DH, Rhee CH. Local tumor control after retreatment of spinal metastasis using stereotactic body radiotherapy; comparison with initial treatment group. Acta Oncol. 2012 May;51(5):589–95.

8. Choi CYH, Adler JR, Gibbs IC, Chang SD, Jackson PS, Minn AY, et al. Stereotactic Radiosurgery for Treatment of Spinal Metastases Recurring in Close Proximity to Previously Irradiated Spinal Cord. Int J Radiat Oncol. 2010 Oct;78(2):499–506.

9. Gagnon GJ, Henderson FC, Gehan EA, Sanford D, Collins BT, Moulds JC, et al. Cyberknife radiosurgery for breast cancer spine metastases: A matched-pair analysis. Cancer. 2007 Oct 15;110(8):1796–802.

10. Garg AK, Shiu AS, Yang J, Wang X-S, Allen P, Brown BW, et al. Phase 1/2 trial of single-session stereotactic body radiotherapy for previously unirradiated spinal metastases. Cancer. 2012 Oct 15;118(20):5069–77.

11. Germano IM, Carai A, Pawha P, Blacksburg S, Lo Y-C, Green S. Clinical outcome of vertebral compression fracture after single fraction spine radiosurgery for spinal metastases. Clin Exp Metastasis. 2016 Feb;33(2):143–9.

12. Gerszten PC, Germanwala A, Burton SA, Welch WC, Ozhasoglu C, Vogel WJ. Combination kyphoplasty and spinal radiosurgery: a new treatment paradigm for pathological fractures. J Neurosurg Spine. 2005;3(4):296–301.

13. Gerszten PC, Burton SA, Ozhasoglu C, Welch WC. Radiosurgery for spinal metastases: clinical experience in 500 cases from a single institution. Spine. 2007 Jan;32(2):193–9.

14. Gibbs IC, Kamnerdsupaphon P, Ryu M-R, Dodd R, Kiernan M, Chang SD, et al. Image-guided robotic radiosurgery for spinal metastases. Radiother Oncol. 2007 Feb;82(2):185–90.

15. Heron DE, Rajagopalan MS, Stone B, Burton S, Gerszten PC, Dong X, et al. Single-session and multisession CyberKnife radiosurgery for spine metastases—University of Pittsburgh and Georgetown University experience: Clinical article. J Neurosurg Spine. 2012;17(1):11–18.

16. Hsu S-W, Chao H-L, Lin K-T, Chou Y-C, Lo C-H, Lee S-Y, et al. Pain relief following spinal lesion treatment with stereotactic radiosurgery: Clinical experience in 65 cases. J Med Sci. 2015;35(4):162.

17. Hunter GK, Balagamwala EH, Koyfman SA, Bledsoe T, Sheplan LJ, Reddy CA, et al. The efficacy of external beam radiotherapy and stereotactic body radiotherapy for painful spinal metastases from renal cell carcinoma. Pract Radiat Oncol. 2012 Oct;2(4):e95–100.

18. Ryu S, Pugh SL, Gerszten PC, Yin F-F, Timmerman RD, Hitchcock YJ, et al. RTOG 0631 phase 2/3 study of image guided stereotactic radiosurgery for localized (1-3) spine metastases: Phase 2 results. Pract Radiat Oncol. 2014 Mar;4(2):76–81.

19. Hwang YJ. Follow-up CT and MR findings of osteoblastic spinal metastatic lesions after stereotactic radiotherapy. Jpn J Radiol. 2012 Jul;30(6):492–8.

20. Jhaveri PM, Teh BS, Paulino AC, Blanco AI, Lo SS, Butler EB, et al. A dose-response relationship for time to bone pain resolution after stereotactic body radiotherapy (SBRT) for renal cell carcinoma (RCC) bony metastases. Acta Oncol. 2012 May;51(5):584–8.

21. Kim. Stereotactic Body Radiotherapy with Helical Tomotherapy for Pain Palliation in Spine Metastasis. Technol Cancer Res Treat [Internet]. 2013 [cited 2017 Jan 16]; Available from: http://tct.sagepub.com/lookup/doi/10.7785/tcrt.2012.500329

22. Księżniak-Baran D, Blamek S, Roch-Zniszczoł A, Stąpór-Fudzińska M, Miszczyk L. Evaluation of efficacy and safety of robotic stereotactic body radiosurgery and hypofractionated stereotactic radiotherapy for vertebral metastases. Współczesna Onkol. 2015;4:327–32.

23. Lee S, Chun M. Pain relief by Cyberknife radiosurgery for spinal metastasis. Tumori. 2012;98(2):238–42.

24. Lee DS, Kwak YK, Jeong SM, Song JH, Kang YN, Lee SN, et al. High-dose Radiotherapy Using Helical Tomotherapy for Vertebral Metastasis: Early Clinical Outcomes and Cord Dose Specification. Jpn J Clin Oncol. 2013 Jun 1;43(6):646–53.

25. Lee E, Kim TG, Park HC, Yu JI, Lim DH, Nam H, et al. Clinical outcomes of stereotactic body radiotherapy for spinal metastases from hepatocellular carcinoma. Radiat Oncol J. 2015;33(3):217.

26. Lee SH, Lee KC, Choi J, Ahn SH, Lee SH, Sung KH, et al. Clinical applicability of biologically effective dose calculation for spinal cord in fractionated spine stereotactic body radiation therapy. Radiol Oncol [Internet]. 2015 Jan 1 [cited 2017 Jan 16];49(2). Available from: http://www.degruyter.com/view/j/raon.2015.49.issue-2/raon-2015-0008/raon-2015-0008.xml

27. Mahadevan A, Floyd S, Wong E, Jeyapalan S, Groff M, Kasper E. Stereotactic Body Radiotherapy Reirradiation for Recurrent Epidural Spinal Metastases. Int J Radiat Oncol. 2011 Dec;81(5):1500–5.

28. Massicotte E, Foote M, Reddy R, Sahgal A. Minimal access spine surgery (MASS) for decompression and stabilization performed as an out-patient procedure for metastatic spinal tumours followed by spine stereotactic body radiotherapy (SBRT): first report of technique and preliminary outcomes. Technol Cancer Res Treat. 2012;11(1):15–25.

29. Muacevic A, Kufeld M, Rist C, Wowra B, Stief C, Staehler M. Safety and feasibility of image-guided robotic radiosurgery for patients with limited bone metastases of prostate cancer. Urol Oncol Semin Orig Investig. 2013 May;31(4):455–60.

30. Napieralska A, Miszczyk L, Stapor-Fudzinska M. CyberKnife stereotactic radiosurgery and stereotactic ablative radiation therapy of patients with prostate cancer bone metastases. Neoplasma [Internet]. 2016 Jan 16 [cited 2019 Jan 2]; Available from: http://www.elis.sk/index.php?page=shop.product_details&flypage=flypage.tpl&product_id=4581&category_id=85&option=com_virtuemart

31. Owen D, Laack NN, Mayo CS, Garces YI, Park SS, Bauer HJ, et al. Outcomes and toxicities of stereotactic body radiation therapy for non-spine bone oligometastases. Pract Radiat Oncol. 2014;4(2):e143–9.

32. Park HJ, Kim HJ, Won J-H, Lee SC, Chang AR. Stereotactic Body Radiotherapy (SBRT) for Spinal Metastases: Who Will Benefit the Most from SBRT? Technol Cancer Res Treat. 2015 Apr;14(2):159–67.

33. Ryu S, Jin R, Jin J-Y, Chen Q, Rock J, Anderson J, et al. Pain Control by Image-Guided Radiosurgery for Solitary Spinal Metastasis. J Pain Symptom Manage. 2008 Mar;35(3):292–8.

34. Schipani S, Wen W, Jin J-Y, Kim JK, Ryu S. Spine Radiosurgery: A Dosimetric Analysis in 124 Patients Who Received 18 Gy. Int J Radiat Oncol. 2012 Dec;84(5):e571–6.

35. Sheehan JP, Shaffrey CI, Schlesinger D, Williams BJ, Arlet V, Larner J. Radiosurgery in the treatment of spinal metastases: tumor control, survival, and quality of life after helical tomotherapy. Neurosurgery. 2009 Dec;65(6):1052–61; discussion 1061-1062.

36. Sohn S, Chung CK, Sohn MJ, Chang U-K, Kim SH, Kim J, et al. Stereotactic radiosurgery compared with external radiation therapy as a primary treatment in spine metastasis from renal cell carcinoma: a multicenter, matched-pair study. J Neurooncol. 2014 Aug;119(1):121–8.

37. Sohn S, Chung CK, Sohn MJ, Kim SH, Kim J, Park E. Radiosurgery Compared with External Radiation Therapy as a Primary Treatment in Spine Metastasis from Hepatocellular Carcinoma : A Multicenter, Matched-Pair Study. J Korean Neurosurg Soc. 2015;59(1):37.

38. Staehler M, Haseke N, Nuhn P, Tüllmann C, Karl A, Siebels M, et al. Simultaneous anti-angiogenic therapy and single-fraction radiosurgery in clinically relevant metastases from renal cell carcinoma. BJU Int. 2011 Sep 1;108(5):673–8.

39. Tsai J-T, Lin J-W, Chiu W-T, Chu W-C. Assessment of image-guided CyberKnife@ radiosurgery for metastatic spine tumors. J Neurooncol. 2009 Aug;94(1):119–27.

40. Wang XS, Rhines LD, Shiu AS, Yang JN, Selek U, Gning I, et al. Stereotactic body radiation therapy for management of spinal metastases in patients without spinal cord compression: a phase 1–2 trial. Lancet Oncol. 2012;13(4):395–402.

41. Ahmed KA, Stauder MC, Miller RC, Bauer HJ, Rose PS, Olivier KR, et al. Stereotactic Body Radiation Therapy in Spinal Metastases. Int J Radiat Oncol. 2012 Apr;82(5):e803–9.

42. Al-Omair A, Masucci L, Masson-Cote L, Campbell M, Atenafu EG, Parent A, et al. Surgical resection of epidural disease improves local control following postoperative spine stereotactic body radiotherapy. Neuro-Oncol. 2013 Oct;15(10):1413–9.

43. Bahig H, Simard D, Létourneau L, Wong P, Roberge D, Filion E, et al. A Study of Pseudoprogression After Spine Stereotactic Body Radiation Therapy. Int J Radiat Oncol. 2016 Nov;96(4):848–56.

44. Bishop AJ, Tao R, Rebueno NC, Christensen EN, Allen PK, Wang XA, et al. Outcomes for Spine Stereotactic Body Radiation Therapy and an Analysis of Predictors of Local Recurrence. Int J Radiat Oncol. 2015 Aug;92(5):1016–26.

45. Chang JH, Gandhidasan S, Finnigan R, Whalley D, Nair R, Herschtal A, et al. Stereotactic Ablative Body Radiotherapy for the Treatment of Spinal Oligometastases. Clin Oncol [Internet]. 2017 Feb [cited 2017 May 2]; Available from: http://linkinghub.elsevier.com/retrieve/pii/S0936655517300791

46. Colaco RJ, Park HS, Laurans MS, Chiang VS, Yu JB, Husain ZA. Spine Stereotactic Body Radiotherapy Outcomes in Patients with Concurrent Brain Metastases. Cureus [Internet]. 2016 [cited 2017 May 2];8(7). Available from: https://www.ncbi.nlm.nih.gov/pmc/articles/PMC4985044/

47. Deodato F, Cilla S, Macchia G, Caravatta L, Mignogna S, Massaccesi M, et al. Extracranial radiosurgery with volumetric modulated arc therapy: Feasibility evaluation of a phase I trial. Oncol Lett. 2013 Jun;5(6):1889–96.

48. Gill B, Oermann E, Ju A, Suy S, Yu X, Rabin J, et al. Fiducial-free CyberKnife stereotactic body radiation therapy (SBRT) for single vertebral body metastases: acceptable local control and normal tissue tolerance with 5 fraction approach. Front Oncol [Internet]. 2012 Apr 26 [cited 2017 Jan 10];2. Available from: http://www.ncbi.nlm.nih.gov/pmc/articles/PMC3355827/

49. Hamilton AJ, Lulu BA, Fosmire H, Stea B, Cassady JR. Preliminary clinical experience with linear accelerator-based spinal stereotactic radiosurgery. Neurosurgery. 1995 Feb;36(2):311–9.

50. Jahanshahi P, Nasr N, Unger K, Batouli A, Gagnon GJ. Malignant melanoma and radiotherapy: past myths, excellent local control in 146 studied lesions at Georgetown University, and improving future management. Front Oncol [Internet]. 2012 [cited 2017 Jan 16];2. Available from: http://journal.frontiersin.org/article/10.3389/fonc.2012.00167/abstract

51. Laufer I, Iorgulescu JB, Chapman T, Lis E, Shi W, Zhang Z, et al. Local disease control for spinal metastases following "separation surgery" and adjuvant hypofractionated or high-dose single-fraction stereotactic radiosurgery: outcome analysis in 186 patients: Clinical article. J Neurosurg Spine. 2013;18(3):207–214.

52. McDonald R, Probyn L, Poon I, Erler D, Brotherston D, Soliman H, et al. Tumor Response After Stereotactic Body Radiation Therapy to Nonspine Bone Metastases: An Evaluation of Response Criteria. Int J Radiat Oncol. 2015 Nov;93(4):879–81.

53. Miller JA, Balagamwala EH, Angelov L, Suh JH, Rini B, Garcia JA, et al. Spine stereotactic radiosurgery with concurrent tyrosine kinase inhibitors for metastatic renal cell carcinoma. J Neurosurg Spine. 2016 Dec;25(6):766–74.

54. Nikolajek K, Kufeld M, Muacevic A, Wowra B, Niyazi M, Ganswindt U. Spinal radiosurgery-efficacy and safety after prior conventional radiotherapy. Radiat Oncol. 2011;6(1):1.

55. Sahgal A, Ames C, Chou D, Ma L, Huang K, Xu W, et al. Stereotactic Body Radiotherapy Is Effective Salvage Therapy for Patients With Prior Radiation of Spinal Metastases. Int J Radiat Oncol. 2009 Jul;74(3):723–31.

56. Thibault I, Al-Omair A, Masucci GL, Masson-Côté L, Lochray F, Korol R, et al. Spine stereotactic body radiotherapy for renal cell cancer spinal metastases: analysis of outcomes and risk of vertebral compression fracture: Clinical article. J Neurosurg Spine. 2014;21(5):711–718.

57. Thibault I, Campbell M, Tseng C-L, et al. Salvage Stereotactic Body Radiotherapy (SBRT) Following In-Field Failure of Initial SBRT for Spinal Metastases. Int J Radiat Oncol. 2015 Oct;93(2):353–60.

58. Ursino S, Montrone S, Cantarella M et al. Stereotactic body radiotherapy of bone metastases in oligometastatic disease: prognostic factors of oncologic outcomes. *Tumori*. 2016;102(1):59-64.

59. Yamada Y, Katsoulakis E, Laufer I, Lovelock M, Barzilai O, McLaughlin LA, et al. The impact of histology and delivered dose on local control of spinal metastases treated with stereotactic radiosurgery. Neurosurg Focus. 2017 Jan;42(1):E6.
